# Neurons under genetic control: What are the next steps towards the treatment of movement disorders?

**DOI:** 10.1016/j.csbj.2020.11.012

**Published:** 2020-11-21

**Authors:** Marian Tsanov

**Affiliations:** School of Medicine, University College Dublin, Ireland

**Keywords:** Movement disorders, Parkinson’s disease, Huntington’s disease, Deep brain stimulation, Optogenetics, Gene therapy, Antisense nucleotides

## Abstract

Since the implementation of deep-brain stimulation as a therapy for movement disorders, there has been little progress in the clinical application of novel alternative treatments. Movement disorders are a group of neurological conditions, which are characterised with impairment of voluntary movement and share similar anatomical loci across the basal ganglia. The focus of the current review is on Parkinson’s disease and Huntington’s disease as they are the most investigated hypokinetic and hyperkinetic movement disorders, respectively. The last decade has seen enormous advances in the development of laboratory techniques that control neuronal activity. The two major ways to genetically control the neuronal function are: 1) expression of light-sensitive proteins that allow for the optogenetic control of the neuronal spiking and 2) expression or suppression of genes that control the transcription and translation of proteins. However, the translation of these methodologies from the laboratories into the clinics still faces significant challenges. The article summarizes the latest developments in optogenetics and gene therapy. Here, I compare the physiological mechanisms of established electrical deep brain stimulation to the experimental optogenetical deep brain stimulation. I compare also the advantages of DNA- and RNA-based techniques for gene therapy of familial movement disorders. I highlight the benefits and the major issues of each technique and I discuss the translational potential and clinical feasibility of optogenetic stimulation and gene expression control. The review emphasises recent technical breakthroughs that could initiate a notable leap in the treatment of movement disorders.

## Graphical abstract

**Figure:**
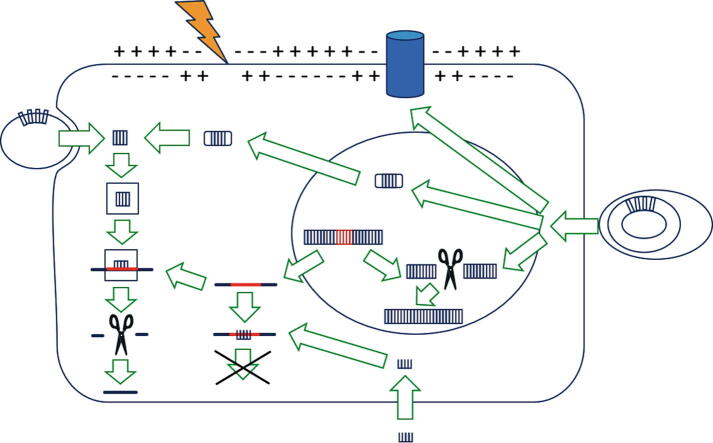

## 1 Introduction

The steps for the treatment of movement disorders seemingly resemble the steps of a patient with Parkinson’s Disease (PD): the initiation of a movement could be more difficult than its execution. If a PD patient overcomes the initial freeze, a movement towards a particular goal could be successfully achieved. Similarly, the therapies for movement disorders face difficulty with the translation of novel solutions from basic neuroscience level, but once translated clinically, their implementation is usually efficient and lasting. The major steps of the fundamental PD research so far were triggered by levodopa pharmacological application, apomorphine infusion pumps and deep-brain stimulation (DBS). Since then the translational advance of PD therapy has stalled in a gait freeze with little development. Now, this is about to change and recent research advances might soon revolutionize the treatment for some neurological diseases. PD is just one representative of the movement disorders, which are a large group of hypokinetic and hyperkinetic conditions with different genesis, symptoms and progression. However, the majority of them share the functional principles of motor execution, their physiology undergoes similar neuromodulatory control and the anatomy of their neuronal circuits generally overlaps. Therefore, novel research findings and treatment innovations for a particular dysfunction would have a noteworthy impact on the therapy for the rest of the movement disorders. The review focuses particularly on the therapeutic approaches designed to control the neuronal function. Other ‘non-neuronal’ therapeutic directions such as immunotherapy and infusion therapy are outside the scope of this review. Immunotherapy uses antibodies and inhibitors that bind to the neurotoxic antigens. Infusion therapies involve continuous application of pharmacological agents such as levodopa and dopamine agonists. Immuno- and infusion therapies are highly-beneficial and may further exert a significant therapeutic impact. The extensive literature about their translation requires a separate review. Here, I will address how the genetic control of neuronal function can be utilized for the treatment of movement disorders. I will review the development of optogenetic DBS and the progress of DNA- and RNA-based gene therapy. The review encompasses the innovative research in the fields of DBS, optogenetics and gene therapy and examines the proximity of the novel methodologies to clinical translation.

## 2 Selective neurostimulation: Can optogenetics change DBS?

The last prominent therapeutic innovation of movement disorders was achieved in the 1980 s [1], [2] after decades of experimental work [3]. It took another decade for the approval of DBS issued by the US Food and Drug Administration (FDA) for the therapy of essential tremor in 1997 [4]. DBS revolutionized the treatment for patients with Parkinson’s Disease (PD) and is currently the gold standard for the treatment of primary motor manifestations of PD in patients with pharmacologically-induced motor fluctuations and dyskinesias [5], [6], [7], [8]. The major benefit of DBS is the smoothing of motor fluctuations, amelioration of the early wearing off, sudden on/off phenomena and involuntary hyperkinetic movements caused by levodopa treatment [9], [10]. DBS was successfully applied for the treatment of other movement disorders such as primary dystonia [11], tardive dyskinesia [12], chorea-acanthocytosis [13], dystonia–choreoathetosis in cerebral palsy [14] and essential tremor [15]. For patients with Huntington’s diseases (HD) the application of DBS to the pallidum has been restricted to a few recent case reports [16]. DBS is a symptomatic treatment and it is limited to the treatment of motor symptoms. Concurrently, DBS has little effect or could even exert an adverse effect for symptoms such as gait, speech or cognitive problems [17], [18]. Another limitation of DBS is that it does not protect against the development of neurodegeneration and this supportive therapy cannot not halt disease progression [8], [19]. The methodological advances in neuroscience and genetics over the last decade indicated that DBS technique can be fundamentally changed through genetic control of the neuronal activity.

DBS is a technique that delivers electric current to basal ganglia regions such as globus pallidus internus, globus pallidus externus or subthalamic nucleus. The neuronal stimulation through electric current is already outdated in the majority of laboratories conducting fundamental neuroscience research. Current-induced stimulation is now widely replaced by light-induced stimulation, a technique which is known as optogenetics [20]. Through optogenetics the neurons are genetically engineered to express light-sensitive proteins known as opsins, which control the flow of ions through the cellular membrane in response to light [21]. Such genetic reprogramming is triggered after an infection by viruses containing genetic construct that carry the opsin gene, along with genetic promoter that controls the opsin expression for specific cell type. The most common opsin is channelrhodopsin (ChR2), a transmembrane protein derived from the green algae Chlamydomonas. This opsin contains a chromophore which, upon absorption of blue light, undergoes a conformational change that causes the transmembrane channel to open, leading to neuronal depolarization and generation of action potentials. The most common groups of viruses used in laboratory environment for cell-specific opsin expression are lentiviral, herpes simplex and adeno-associated viruses (AAV) [22]. The opsin-coding virus must be injected in the brain structure of interest and for depolarization or hyperpolarization of the infected cells they must be illuminated with laser light in the wave-length spectrum that activates the expressed opsin [23].

The three main advantages of light-induced neuronal stimulation that position it as the preferred lab stimulation technique are: 1) physiological depolarization, 2) neurotransmission specificity and 3) neuronal selectivity. The physiological depolarization relies on threshold-dependent depolarization of the axonal hillock followed by axonal action potential with orthodromic spike propagation. This is compromised in electrically-evoked depolarization where the electric field depolarizes simultaneously different segments of the neurons, leading to antidromic spike propagation. This disadvantage of electrical stimulation leads to possible recruitment of distant neurons through direct axonal stimulation [24]. Simultaneous depolarisation of different cell bodies, dendrites and axonal projections results in non-physiological activation of the neuronal populations that can disturb the connectivity between neurons and their synaptic plasticity. While the electrical stimulation depolarizes simultaneously excitatory and inhibitory neurons, the optogenetic stimulation depolarizes either excitatory or inhibitory neurons, protecting the balance between excitation and inhibition. Optogenetic neurotransmission-specific activation preserves fundamental network properties such as feed-back inhibition and feed-forward inhibition, lateral inhibition, counter inhibition, recurrent excitation, divergence and convergence of signal propagation [25], [26]. In comparison, the non-specific effect of electric current triggers disproportional response of individual neurons with overall decreased network response [27], [28]. The key advantage of optogenetics is the neuronal selectivity: stimulation of particular type of neurons that express the promoter encoded in the viral vector [29], [30].

## 3 Electrical vs optogenetic stimulation: Can we solve the puzzle of DBS?

Currently, the most effective treatment for advanced PD is the electrical DBS with electrodes implanted in the subthalamic nucleus or internal globus pallidus, and with frequency of the electrical current in the range of 120–130 Hz, delivered continuously. It is not fully understood what makes this stimulation protocol so efficient. The efficiency of DBS is a matter of controversy [31], [32] and more likely to result from combination of physiological excitation and inhibition, superimposed by non-physiological disruption of the neuronal activity [33]. The complex mechanism of DBS is further complicated by the heterogeneity of the neuronal tissue that is affected by the electric current, where the tissue is composed of different neuronal groups and axonal projections [32], [34]. While the underlying mechanism of DBS is still a matter of debate there are three major hypotheses: inhibition hypothesis, excitation hypothesis and disruption hypothesis according to which DBS abnormally disrupts in non-physiological manner the synaptic transmission [35], [36]. It is also possible that neuronal inhibition, excitation and disruption simultaneously contribute to DBS [37], [38]. Electrical DBS of globus pallidus internus has therapeutic effects to PD and dystonia, affecting the motor symptoms in both diseases in a manner similar to lesion therapy. This led to the concept that DBS interrupts abnormal information flow via functional disconnection of the stimulated neuronal structures [39], [40], [41]. The disruption of pathological nigro-striatal circuitry by DBS is proposed to occur at the molecular, cellular and network levels [42], [43]. It is proposed that aberrant DBS stimulation leads to synaptic filtering of abnormal signal processing in the basal ganglia of patients with PD and dystonia [44], [45]. The hypothesis that DBS disrupts the abnormal neuronal activity on population level was supported by the observation that some symptoms of PD and dystonia are associated with pathological synchronization of neural populations [46]. Coordinated reset stimulation [47] was developed to specifically desynchronize abnormally synchronized oscillations [48], [49]. This approach was designed to deliver phase resetting stimuli at different times to different sub-populations involved in abnormal neuronal synchronization [47].

The classical model predicts that deactivation of subthalamic nucleus (STN) halts the dysfunctional excitatory subthalamic activity, which is abnormally increased in hypokinetic- (PD) and abnormally decreased for hyperkinetic movement disorders (HD and dystonia) [50], [51], [52]. The dysfunction of direct and hyperdirect pathways in hypokinetic movement disorders reduces the disinhibition in thalamus and cortex, while the dysfunction of hyperdirect and indirect pathways in hyperkinetic disorders increases the thalamic and cortical disinhibition [53], [54], [55], [56]. Subsequent optogenetic research established that this model is oversimplified. Optogenetic stimulation of the direct and indirect pathway projection neurons evoked heterogenic postsynaptic response and diverse cellular effects in substantia nigra neurons, with stimulation of each pathway eliciting both excitations and inhibitions [57]. Concurrent activation of the spiny projection neurons of both pathways preceded the initiation of contraversive movements [58]. Seminal review proposed that the direct and indirect pathways communicate via complex interneuronal network and the communication between the neuronal subtypes within the basal ganglia may functionally bridge the two pathways [59]. The concurrent physiological activation of both pathways during movement suggests that the lack of coordinated activity between these two pathways may be related to the bradykinesia, freezing and gait festination in PD patients [59], [60]. Control of distinct circuit elements in parkinsonian rodents in a fundamental study by Gradinaru et al revealed that the effect of DBS therapy may be mediated by different projections within the basal ganglia pathways [61]. PD model of rats did not show even minimal changes in rotational behaviour after optical inhibition of the local excitatory STN neurons. High-frequency stimulation delivered locally to the STN also failed to affect PD symptoms [61]. However, high-frequency stimulation of afferent projections to the STN in mice robustly and reversibly ameliorated PD symptoms, measured by rotational behaviour and head position bias. High-frequency activation of layer V motor cortex projection neurons also succeeded to ameliorate PD symptoms in a manner similar to that of STN. The authors concluded that direct cellular inhibition or stimulation or STN had little or no effect, while stimulation of the afferent fibers to STN substantially improved the PD symptoms [61]. Another study showed that excitation of medium spiny projection neurons in the indirect-pathway elicited a PD-like symptoms such as increased freezing, bradykinesia and decreased locomotion, while activation of direct pathway rescued PD-like deficits with decreased freezing and increased locomotion of a mouse model of PD [62]. Photoinhibition of rebound firing of ventrolateral thalamic neurones has been also shown to reduce the tremor and rigidity in a mouse model of PD [63]. Optogenetic interventions that dissociate the activity of two neuronal populations in the external globus pallidus restored movement in dopamine depleted mice for several hours after the stimulation [64]. While optogenetic stimulation of a particular circuitry is able to ameliorate the movement symptoms, stimulation of a single pathway is unable to address other parkinsonian symptoms such as postural and gait symptoms [64]. Optogenetic studies in rodent models of Huntington’s disease showed that multiple sources within basal ganglia circuitry contribute to increased inhibitory control on medium-sized spiny neurons of the indirect pathway [65], [66].

The translational implication arising from these studies is that optogenetic stimulation must target different neuronal types and pathways to address the full spectrum of symptoms in patients with movement disorders. The optogenetic DBS may need to target more than one cell population for the parallel treatment of motor, postural and gait symptoms. The cognitive and speech symptoms observed in movement disorders will require the targeting of additional brain networks and their treatment unlikely will be addressed by the first translated generation of optogenetics DBS. Whether the optogenetic physiological stimulation will translate into better therapeutic treatment of movement disorders remains an open question. The translation of optogenetic methodology requires: 1) identification of the most suitable neuronal population or pathway that can mediate the therapeutic effect of DBS, 2) identification of the most efficient stimulation or inhibition frequency. While for some movement disorders, such as PD, the neuronal groups and stimulation protocol for DBS have been identified, for others, such as HD, it will take substantial pre-clinical experimental work before clinical trials are initiated.

## 4 Brain-computer interface for DBS

**Fig. 1:**
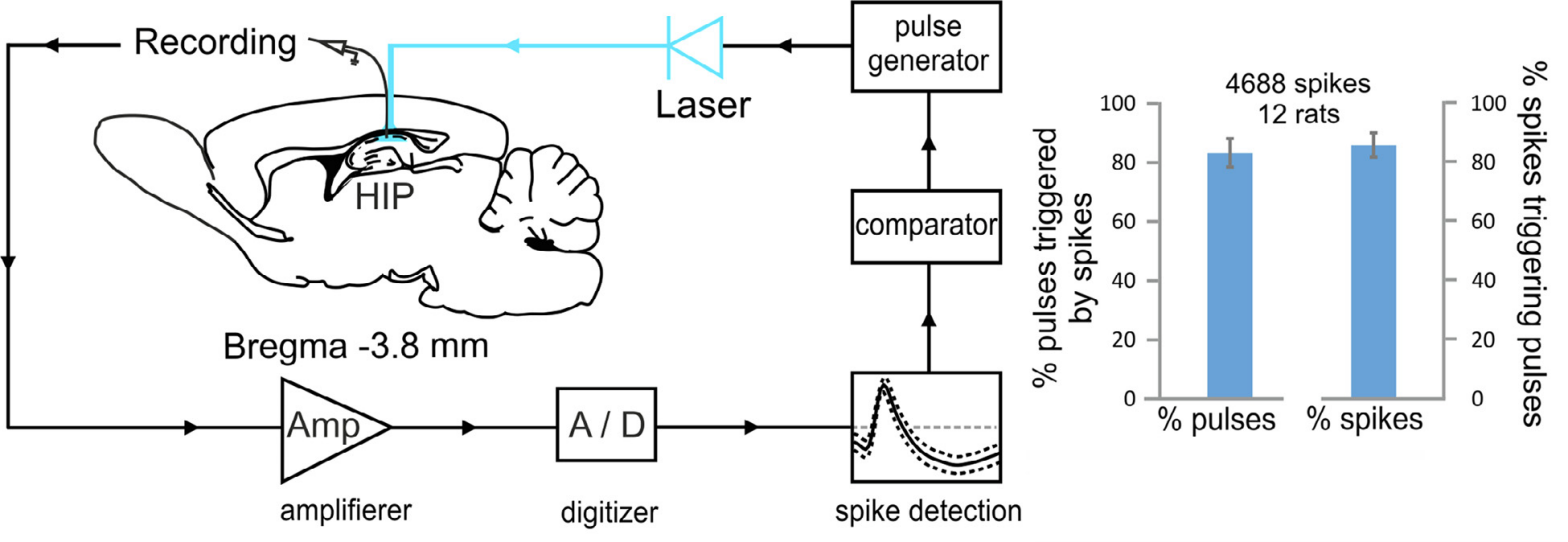
Optogenetic closed-loop brain-computer interface Left: general schematics of optogenetic closed-loop brain-computer interface (BCI). The recording and stimulation are located in the hippocampus (HIP). Right: percent of pulses triggered by the spikes of the selected hippocampal place cells, excluding recording artifacts of electrical noise and spikes from other neurons (left bar) and percent of spikes from the selected place cells that accounted for the triggered pulses, excluding the unsuccessfully detected spikes (right bar). Adopted from [71], [188].

The translational future of optogenetic DBS became even brighter after another technical development: the brain-computer interface (BCI). Closed-loop BCI interface allows the patterns of neuronal recordings to control the external stimulation [67], [68]. This is achieved by extracellular recording where the electrode is placed in close proximity to a group of neurons to pick up their spiking activity [69], [70]. The detection algorithm for spike sorting and the pulse generation together lead to delay in the BCI circuitry. For efficient optogenetic closed-loop BCI the detection of the spikes must be followed by light pulses with minimal time delay in the range of milliseconds (Fig. 1). Recent preclinical tests of optogenetic BCI in rats showed that the delay can be limited to few milliseconds [71]. The total BCI circuitry delay was 6.3–7.3 ms, including the 5 ms pulse duration, and this was within the spike-timing dependent plasticity window of 40 ms [72], [73]. The methodological development of BCI circuitry has two major goals: 1) to decrease the number of stimulation pulses triggered by recording artifacts including electrical noise and spikes from other neurons that are not the target of investigation, and 2) to decrease the number spikes from the neurons of interest that fail to trigger of stimulation pulses. Recent development reached a rate of less than 20% for both parameters [71]. This BCI design reached a degree of spike detection of 82.1 ± 4.4% (Fig. 1), which was higher than previous report on limbic closed-loop BCI with 52.9 ± 9.5% spike detection success rate [74].

Desynchronizing feedback stimulation approach is suggested to be effective closed-loop technique for the control of abnormally-synchronized neuronal populations observed in movement disorders [75], [76]. Closed-loop DBS was performed in pre-clinical research and clinical trials with recording of local field oscillations. The power level of beta range (12–38 Hz) of the local field potential recorded in the subthalamic nucleus of patients with Parkinson’s disease is sufficient to trigger DBS via closed-loop BCI [77]. Closed-loop DBS paradigms triggered by pathological oscillatory activity rather than neuronal spiking is proposed as effective management of advanced PD [78]. Adaptive DBS, consisting of closed-loop, real-time changing of stimulation parameters according to the patient's clinical state, offers patient-centred treatment of movement disorders with reduction of the negative side effects [79]. Closed-loop BCI adaptive DBS achieved a 56% reduction in stimulation time compared to conventional continuous DBS, with a parallel decrease in energy requirements [80]. The authors of this clinical study showed that motor scores from patients with advanced PD improved by 66% (unblinded) and 50% (blinded) during closed-loop BCI adaptive DBS, which was 29% and 27% better than conventional continuous DBS, respectively [80]. Another frequency range was also detected as a reliable signal for closed-loop DBS for movement disorders. A distinctive narrowband gamma oscillation (60–90 Hz) associated with dyskinesia was recognized in the human motor cortex [81]. The authors found that this frequency range was associated with dyskinesia that occurs following medication alone and with dyskinesia which occur during DBS [81]. Subsequent study examined adaptive DBS using the feedback of gamma frequency for closed-loop DBS in patients with PD [82]. The authors used the gamma cortical oscillation in the range of 60–90 Hz, associated with dyskinesia, to decrease stimulation voltage when gamma oscillatory activity was high and increase stimulation voltage when it was low [82]. This approach saved about 40% of the DBS device’s battery energy from traditional constant stimulation. The researchers found that the adaptive approach was at least as effective at controlling symptoms as constant stimulation, but further clinical trials were needed to evaluate the clinical benefits.

Despite the promising start of adaptive DBS, this approach has been restricted to limited clinical trials and further technical development is needed for the approval of closed-loop DBS as a validated treatment option. The translation of closed-loop BCI adaptive DBS will require: 1) improvement of the signal detection algorithms, 2) development of electrophysiological biomarkers for the detection of dysfunctional neuronal activity across different movement disorders. The resolution of these issues will take several years and large number of clinical trials for the optimization of this methodology. The combination of DBS and BCI can advance the therapy of movement disorders, while the combination of optogenetics and BCI may lead to ground-breaking patient-centred therapy [83]. The optogenetic-based DBS solely depends on the application of adeno-associated viruses in humans.

## 5 Successful translation of adeno-associated virus transfection

Adeno-associated virus (AAV), which is the most common virus used in optogenetics, is safe for use in rodents [20], [84] and non-human primates [85], [86]. The translation of AAV injection in the human brain has been faced with concerns about the degree of neurotoxicity and immunological response. When the neuronal genome is artificially reprogrammed to produce large number of proteins such as the light-sensitive opsins this could lead to structural or functional adverse effects. Viral infection and the subsequent overexpression of membrane proteins could potentially change the cellular capacitance and affect the cellular physiology, causing toxicity [87]. The AAV promoters could lead to the expression of opsins at very high levels that can lead to protein accumulations or structural abnormalities of the neurons over time [88]. In the dawn of optogenetics it was considered that that the translation of AAV would take a substantial period of time before being safely applied to humans. However, we know now that AAV is tolerated by the human immune system [89] and shows very low neutralizing factor seroprevalence in humans [90]. Viral vectors are stable in postmitotic cells like neurons and AAV generally show reliable penetrance and diffusion with the brain tissue [91]. The most common strains used in pre-clinical research and clinical application are not genotoxic because they form episomal concatemers that exist outside the host genome [92]. The recombinant AAV particles are safe because they lack any viral genes and contain only DNA sequences designed for therapeutic applications and therefore there is no active viral gene expression to amplify the immune response [93]. Also, AAV have been shown to be less immunogenic than other viruses due to the ability of AAV not to efficiently transduce antigen-presenting cells [94]. Pre-existing immunity to AAV [95] can often be overcome by selecting a specific AAV variant that has not induced memory responses of the immune system involving neutralizing antibodies and T cells [96]. Furthermore, recombinant techniques involving capsid shuffling have been utilized to engage novel AAV variants with reduced sensitivities to neutralizing antibodies [97], [98], [99].

The European Medicines Agency (EMA) approved AAV-based product alipogene tiparvovec (Glybera by uniQuire) [100]. Alipogene tiparvovec is approved gene therapy that aims to reverse the inherited lipoprotein lipase deficiency that leads to pancreatitis. Local administration of AAV in the eye is awaiting approval for RPE65-mediated inherited retinal dystrophy (Voretigene Neparvovec by Spark Therapeutics) [101], [102]. AAV is used to deliver a functioning copy of the human retinal pigment epithelium-specific gene into retinal cells of patients with reduced or absent levels of RPE65 protein [103]. AAV was also surgically delivered in the basal ganglia of patients with movement disorders. After successful human trials AAV transfection was approved for clinical use to treat PD [104]. Gene transfer of glutamic acid decarboxylase (GAD) modulates the production of GABA in the subthalamic nucleus and is believed to improve the basal ganglia function in PD patients. The authors injected bilaterally AAV-GAD in the subthalamic nucleus of PD patients. The AAV-GAD treatment group of patients showed a significantly greater improvement compared to the sham group [104]. The adverse events such as headache or nausea were mild or moderate, likely related to the surgery and resolved. Currently, approximately 60 clinical trials use AAV; recent summary of in the approved clinical applications of AAV is provided by Naso and colleagues [105]. Overall, AAV has been shown to be safe and effective in pre-clinical and clinical settings. The translation of AAV is accomplished for the gene therapy of few disorders, while other candidate disorders await clinical trials for safety and tolerability. However, when it comes to optogenetics the application of AAV needs additional considerations.

## 6 AAV application for optogenetics: Translation under construction

The translation of optogenetics to humans faces other technical challenges such as limitation in the cell specificity for non-transgenic species and variability of opsin expression. Currently, the optogenetic modulation of neural activity in non-transgenic animals and primates is successful only for a few promoters [106]. Cell type selectivity in primates is achieved by viral vectors that carry small promoter sequences, while for several cell types in the basal ganglia, there is no specific promoter that is sufficiently compact to incorporate into the AAV viral vector [106]. AAV can carry DNA genome of up to 5 kilobases (kb), which is a limitation for promoters with high genetic load. The most common promoter for cell-type specific expression of ChR2 in excitatory neurons in primate studies is CaMKIIα [21], [107], [108]. Other promoters used in primates are the ubiquitous promoters human synapsin (hSyn), elongation factor 1α (Ef1α) and human thymocyte-1 (hThy-1) [109], [110], and the pan-cellular promoter CAG [111], [112]. Laboratory research extends the expression specificity to large number of cell types by combining the promoter-based approach with recombinase systems in rodents [113], [114]. The selectivity of optogenetics in rodents relies on several genetic promoters expressed in cre-driver transgenic lines. The expression of ChR2 in neuronal subtypes is accomplished by injecting the brain of transgenic animals with a cre-inducible viral construct embedded in AAV. The transgenic mice and rats express cre-recombinase under the control of the endogenous parvalbumin (PV) or somatostatin (SST) promoters that enables selective expression in PV- or SST-positive interneurons [115]. Optogenetic stimulation of PV-expressing neurons in the globus pallidus externus restores movement in dopamine-depleted mice [64]. Recent finding showed that optogenetic stimulation of cortical SST interneurons was able to alleviate the motor symptoms in parkinsonian mice [116]. The clinical translation of these studies requires a mechanism for optogenetic control of neuronal subtypes such as PV and SST cells in non-transgenic animals. Injection of single AAV vectors in macaque neurons, however, resulted in limited expression of PV-expressing neurons [117]. The expression of AVV in selected neurons of non-transgenic animals requires different approach: co-injection of AVV caring the gene for the cre recombinase under the control cell-type specific promoter with another AVV caring the gene for ChR2 driven by ubiquitous promoter. This approach was successfully achieved for tyrosine hydroxylase (TH)-expressing dopaminergic neurons [118]. The combination of cell-type specificity mediated by the small TH promoter and cre-recombinase-dependent ChR2 expression mediated by the Ef1α promoter allowed the optogenetic activation of TH-positive neurons in the midbrain of wild-type Rhesus macaques. For successful translation of optogenetics for the treatment of different movement disorders, however, we need further pre-clinical research for the selective targeting of neuronal sub-types such as PV- or SST-expressing cells.

**Fig. 2:**
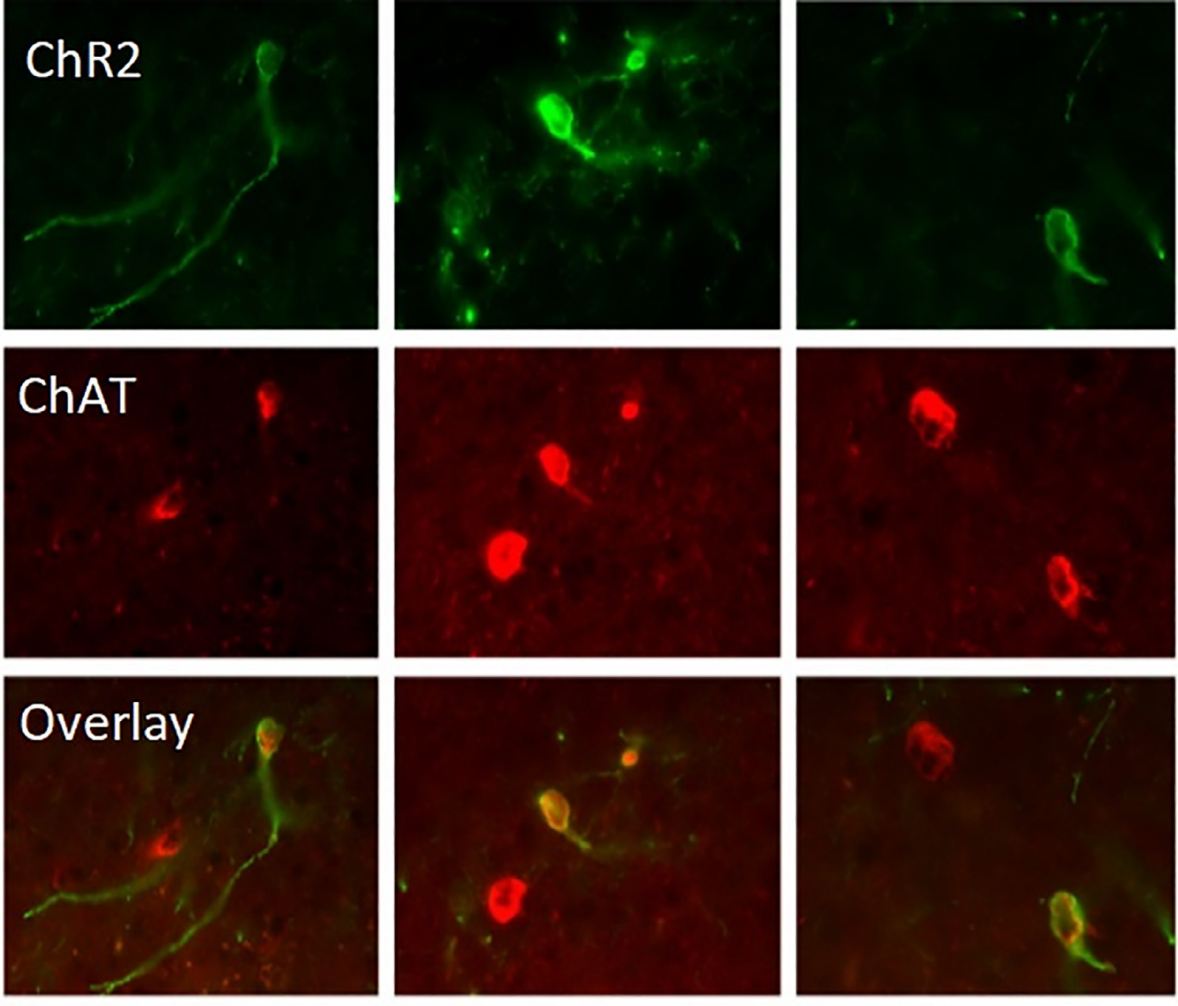
Degree of cell-specific ChR2 expression. Colocalization of channelrhodopsin (ChR2) expression and choline acetyltransferase (ChAT) in the medial septum of ChAT-Cre rats. Top images: the expression of ChR2, shown in green, is visualized by concurrent expression of yellow fluorescent protein (YFP) after injection of cre-dependent virus in the medial septum. Middle images: the detection of ChAT-positive neurons in the same histological preparations, shown in red, after immunohistology for anti-ChAT antibody and fluorescence microscope at 594 nm for the secondary Alexa Fluor antibody with Alexa Fluor 594 nm. Bottom images: co-localization of ChAT and ChR2. Note that in each of the three images there is a ChAT-positive neuron that is not co-expressing ChR2. Adopted from [120]. (For interpretation of the references to colour in this figure legend, the reader is referred to the web version of this article.)

The success rate of opsin expression is another major consideration for the clinical translation of optogenetics. The variability of opsin expression depends on different factors such as the animal species, brain volume, targeted cell types and even technical skills. In transgenic mouse line, expressing cre-recombinase under the choline acetyltransferase (ChAT) promoter, 91.3 ± 1.3% of neurons that expressed yellow fluorescent protein (YFP, which quantifies the degree of ChR2 expression) also stained for the ChAT antibody and 93.5 ± 2.8% of neurons that stained for the ChAT antibody also expressed YFP [119]. This percentage was much lower in transgenic rat line expressing cre-recombinase under the ChAT promoter where 90 ± 5% of neurons that expressed YFP also expressed ChAT, while only 45 ± 5% of neurons that expressed ChAT also expressed YFP [120] (Fig. 2). The variability of opsin expression depends also on the promoter. Compared to the cholinergic neurons, the ChR2 expression in dopaminergic neurons in transgenic mice was lower where ~50% of the neurons in the ventral tegmental area (VTA) of mice that were TH-positive also expressed YFP [121]. The proportion of targeted dopaminergic neurons that expressed this transgene differed in rats. The ChR2 expression in the VTA dopaminergic neurons of transgenic rats showed that 61% ± 4% of neurons that expressed TH also expressed YFP [122]. The opsin expression is highly variable across laboratories even in the same species after injections with the same volume and the same viral titer. The ChR2 expression in the same strain of transgenic TH-cre rats with the same titer of 1.5–8 × 10^12^ particles per mL showed that 52 ± 8% of neurons that expressed TH also expressed YFP in one lab [123], and this result was lower with ~ 10% compared to the result of another lab [122]. These findings indicate that the variability of opsin expression depends even from subtle factors such as the researcher’s technical skills and the commercial batch of the AAV. While such levels of variability among labs are common for the fundamental research, this degree of uncertainty poses a challenge for the clinical translational of optogenetics. Additional research is needed for the translation of AAV for optogenetic-based treatments. The translation of AAV for optogenetic DBS requires: 1) reduction of the expression variability, 2) increase of the number of targeted cell types by combined expression of promoters. The time required for the realization of these steps will depend on the targeted cell types and brain structures of interest but overall, the translation of AAV for optogenetic DBS will be achieved in the next few years.

## 7 Illumination of the brain: Size matters

The optogenetics must also resolve the technical issue of continuous light delivery in large brains. Rodents are much easier target due to their small brain volumes and light can be delivered in the structure of interest by a single optic fiber. The level of penetration of the light within the neuronal tissue shown as a function of distance from the fiber tip in brain tissue [124]. The fraction of light power density decreases due to absorption, scattering, and geometric light spread [88]. The light transmission decreases ~80% at a distance of 1 mm from the optic fiber tip, with exponential decrease of the initial power density [124]. A single optic fiber can efficiently stimulate or inhibit neuronal population of approximately 1 mm^3^ [88] but that radiant flux of the blue and yellow light photons drops to 1% into gray brain matter roughly 1 mm from the fiber tip [125]. This is inconvenient for DBS in the human brain where the size of subthalamic nucleus is approximately 100 mm^3^ [126]. There are two ways around this technical issue for the clinical translation of optogenetics: 1) higher number of fiber tips and 2) longer wavelength. Higher number of light sources can be achieved by implantation of multiple fibers or by optic fibers with a tapered end and these approaches were tested for the illumination of primate brain structures [127], [128]. Continuous exposure to laser light can induce temporary dysfunction of the neuronal activity if the temperature of the tissue is increased over 2 °C; furthermore, increase of 4 °C could lead to permanent damage of the neuronal physiology [129], [130]. Therefore, powerful illumination of large brain structures over several years may induce adverse effects. Another solution is longer light wavelength. Longer-wavelength light is scattering less and penetrating deeper in the brain tissue compared to other visible wavelengths. Red light (635 nm) can be absorbed by the brain tissue 5 times less than the blue light (473 nm) [128]. This allows the red light to illuminate large volume of brain tissue up to 3–5 mm [131]. Thus, the translation of optogenetics is closely dependent on the recent development of opsins activated by red-shifted light: C1V1 [132], Chrimson [133], Jaws [131] and BReaChES [134]. Combination of higher number of fiber tips and light with wavelength in the red spectrum range demonstrated that optogenetics can reach a volume of ~ 10 mm^3^ in the tissue of a macaque brain [128]. The implementation of optogenetics depends on the development of the laser light delivery in the human brain. The translation of optogenetics requires: 1) reduction of the overheating of the neuronal tissue due to prolonged light exposure; 2) improvement of the light distribution in large neuronal structures by implantation of multiple probes or by increase of the wavelength. The optogenetic DBS needs several years of pre-clinical research and several years of clinical trials before being validated as a feasible, safe and reproducible procedure with distinct advantages over the electrical DBS. Until then we should consider alternative ways to revolutionize the therapy of movement disorders.

## 8 Gene therapy: Alternative direction for movement disorders therapy

**Fig. 3:**
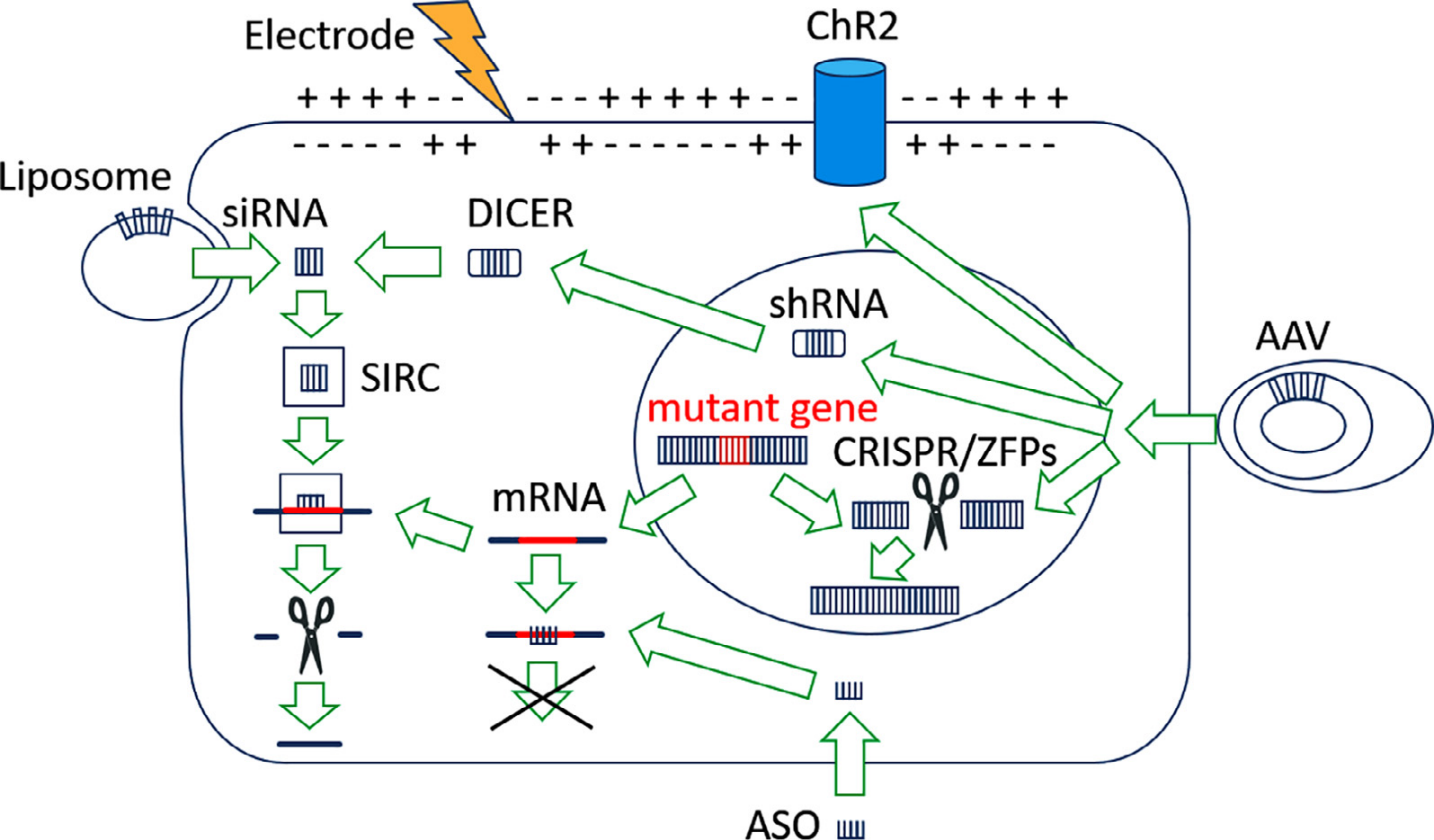
Therapeutic strategies for the treatment of movement disorders. Neuronal control can be exerted by regulation of the depolarization or by regulation of the protein expression. The optogenetics requires the expression of opsins, i.e. channelrhodopsin (ChR2). Optogenetics and gene therapy are delivered through adeno-associated viruses (AAV). DNA-mediated gene editing is represented by CRISPR-cas9 and zinc finger proteins (ZFPs), which are inactivating the mutant gene. The RNA-mediated protein silencing involves three major variations: small interfering RNA (siRNA), short hairpin RNA (shRNA) and antisense oligonucleotides (ASO). shRNA requires enzymatic cleaving by Dicer. Both siRNA and shRNA pathways converge at the RNA-induced silencing complex (RISC) that targets the mutant messenger RNA (mRNA).

The Nobel Prize for Chemistry in 2020 was awarded to Emmanuelle Charpentier and Jennifer Doudna for the development of genome editing methodology. Their finding introduced a new conceptual approach for genetic control of dysfunctional neurons. While the concept of optogenetics is to stimulate populations of neurons, which are physiologically impaired due to advanced neurodegeneration, the concept of genome editing is to genetically reduce or slow down the process of neurodegeneration itself. Instead of injecting the brain with AAV viral vector that carries the gene for opsin expression as it is with optogenetics, gene therapy uses AAV viral vector to edit the mutant genes responsible for neurodegeneration (Fig. 3). The DNA gene editing methodology includes two main techniques: CRISPR-Cas9 and zinc finger proteins (ZFPs). Both techniques use a protein-coding sequence that makes the neurons produce a functional therapeutic protein.

Clustered regularly interspaced short palindromic repeats (CRISPR) and the accompanying CRISPR-associated system (Cas) genome editing constructs is a technique in which guide RNAs direct the nuclease Cas9 to selected sequences of genomic DNA. The enzyme Cas9 is recruited as molecular scissors to cuts both strands at a precise location and repair or remove the mutation. The genomic DNA is restored by non-homologous end joining or homology-directed repair; for current variants of CRISPR-Cas9 see reviews [135], [136]. The benefit of CRISPR-Cas9 is that it could repair mutations at practically any location in genomic DNA [137], [138]. Preclinical experiments showed an impressive efficacy of DNA-based gene therapy and paved the way for subsequent clinical translation. CRISPR-Cas9-mediated editing of germline DNA revealed that this methodology can prevent muscular dystrophy in mice [139]. Subsequent study successfully established CRISPR-Cas9-based genome editing as a potential therapy to treat Duchenne muscular dystrophy [140]. The application of muscle-specific CRISPR-Cas9 dystrophin gene editing was able to ameliorate pathophysiology in a mouse model for Duchenne muscular dystrophy [141]. The authors used AAV viral vectors to edit via CRISPR-Cas9 specific regions of the gene responsible for the Duchenne muscular dystrophy and found that the treated muscles express dystrophin in up to 70% of the myogenic area and increased force [141]. A recent study showed the enormous potential of CRISPR-Cas9 technology as a novel epigenetic-based therapeutic approach for Parkinson’s Disease. The authors used an “all-in-one” lentiviral vector genetic technique for downregulation of SNCA gene expression levels, and the restored the physiological levels of SNCA mRNA allowed the dopaminergic neurons to maintain neuronal function [142]. Preclinical work also described permanent inactivation of Huntington’s disease mutation by allele-specific CRISPR-Cas9 technique in cell culture [143] and in a mouse model of HD [144]. These findings showed that CRISPR-Cas9-mediated excision on the disease chromosome successfully prevented the generation of mutant Huntingtin (htt) mRNA. The application of CRISPR-Cas9 for genome editing is quickly evolving but the clinical translation of this technique for the treatment of movement disorders is in early stages. The experimental design and translational implementation of a nucleotide construct that interacts directly with DNA to supress the transcription of the mutant genes brings particular challenges. One of the main dangers of CRISPR-Cas9 methodology is non-specific editing of the DNA in different genome locations [145]. Therefore, CRISPR/Cas9 may generate a number of nonspecific mutations in the genome [135]. The issue with CRISPR/Cas9 is that accidental changes to the genome would be permanent and can deteriorate other physiological functions of the cells. Another translational concern is the unwanted immune response of the CRISPR-Cas9 methodology [136]. A recent study showed that exogenous proteins such as Cas9 are shown to trigger humoral response and specific antigen T-cells in healthy human volunteers [146].

Another technique for DNA gene therapy is the zinc finger proteins (ZFPs). ZFPs are characterized with zinc finger array that targets selected DNA sequence and each individual finger relates to three bases. ZFPs include zinc finger nucleases known to cleave DNA and zinc finger transcription factors that can regulate gene expression [147]. Zinc-finger nucleases are able to modify the DNA and their binding specificity makes them capable of targeting virtually any gene of interest. ZFPs repressors successfully reduced the mutant htt expression in the brain of a mouse model of HD [148]. Allele-specific repression of mutant htt expression by ZFPs transcriptional repressors was achieved without adversely affecting the expression of other genes, proposing the ZFPs as potential DNA-targeting therapeutic compounds. A recent work revealed the impressive potential of ZFPs for therapeutic use against HD. ZFPs transcription factors, delivered via AAV viral vectors targeted the pathogenic trinucleotide repeat and selectively lowered the mutant htt as a therapeutic strategy [149]. Using patient-derived fibroblasts and neurons, the authors showed that ZFPs selectively supressed >99% of aberrant HD-causing alleles and at the same time preserved the expression of >86% of normal alleles [149]. For clinical success the ZFPs technique must overcome a particular disadvantage: the induction of inflammatory and immune reactions due to the non-native nature of the proteins [150]. This adverse effect can lead to cellular death and restricted duration of the therapeutic effect. There are attempts to overcome this problem. Deimmunization based on host matching of ZFPs was shown to deliver long-term mutant Huntingtin repression in mice without the development of strong side effects [151]. Another major transitional issue is the irreversibility of ZFPs gene therapy. Similarly, to CRISPR-Cas9 the ZFPs therapy cannot be deactivated in case of accidental genetic error [150].

Overall, the successful translation of gene therapy will be able to halt the process of neurodegeneration. This achievement will be able to affect not only the motor symptoms of movement disorders (tremor, rigidity and dyskinesia) but also other symptoms due to dysfunctional brain circuits (gait, speech and cognition). The translation of DNA gene therapy requires additional pre-clinical tests regarding: 1) aversive immunological and inflammatory responses, 2) non-specific gene editing. It is a matter of time that these issues will be resolved but the safety concerns make the forthcoming initiation of this methodology uncertain. While the adverse effects of DNA gene editing can lead to unwanted permanent mutations of the genome, RNA gene therapy, by contrast, allows the researchers to make only temporary regulations of the gene expression. If the RNA-based suppression of mutant genes results in accidental adverse effect it can be easily discontinued. Because the neurons will rapidly degrade the RNA nucleotides, any errors evoked by the RNA-based treatment would be soon washed out. If we are searching for the imminent next step of the gene therapy in patients with movement disorders, we may have to look at RNA technique.

## 9 Is RNA gene therapy closer to clinical translation?

In 2006 Andrew Fire and Craig Mello were awarded with the Nobel Prize for their finding that RNA interference (RNAi) methodology can silence targeted gene expression. Their study demonstrated how RNAi suppresses gene translation, by neutralizing targeted mRNA molecules [152]. The RNAi technique includes two major variations: small interfering RNA (siRNA) and short hairpin RNA (shRNA), for reviews see [153], [154]. Both variations mediate their effect by binding the mRNA of a targeted gene to either block translation or cause degradation of the transcript [155], [156]. One strand of siRNA or micro RNA (miRNA) is incorporated into the RNA-induced silencing complex (RISC) where the sense strand is degraded. The RISC supresses translation of the mRNA, which results in silencing of the dysfunctional gene leading to neurodegeneration (Fig. 3). The dysfunctional gene in HD is the Huntingtin (htt) gene, located in the chromosome 4p16.3 and characterized by excessive trinucleotide CAG repeats. The pathogenic expansion of abnormal polyglutamine tract of the htt protein leads to neurotoxic protein clumping with subsequent neuronal death. PD is a disorder with polygenetic pathology where the most common familial cases of PD are triggered by genetic errors in the LRRK2, PARK7, PINK1, PRKN, or SNCA genes.

Preclinical tests showed enormous potential of the RNAi methodology for the therapy of movement disorders [153]. RNAi improves motor neuropathological abnormalities in mouse [157] and rat [158] models of HD. AAV-mediated RNAi in a mouse model of HD is effective at transducing >80% of the cells in the striatum and partially reducing the levels of both wild-type and mutant htt protein with significant improvement of motor behaviour [159]. One of the major problems of the siRNA therapy is the unwanted immune response leading to side effects and issues with safety and tolerance [160]. If injected in the bloodstream the siRNA accumulates predominantly in the liver, whereas if injected in the cerebrospinal fluid it diffuses poorly within the brain tissue. RNA molecule does not spread sufficiently within brain tissue after intraventricular administration and therefore siRNA administration requires injections of liposomes or polymers [161]. Although the safety and delivery of siRNA have been substantially improved in recent years [162] the delivery procedures and stable expression of RNAi suppression constructs remain major challenges for gene therapy applications [161].

This is not the case with shRNA, which is easily delivered by AAV. The shRNA technique uses a transcribed RNA that is enzymatically cleaved of hairpin by Dicer (Fig. 3). The advantage of shRNA compared to the siRNA technique is that: 1) by integrating in the host genome the shRNA can be continually expressed for months or years, and 2) by using the endogenous processing machinery reduces the risk for off-target effects [156], [163]. AAV-mediated administration of shRNA ameliorated neuropathology and induced partial reversal of disease progression in Huntington disease mice models [164], [165]. Preclinical tests in mice supported by UniQure showed that injection of AAV with micro-RNA in the striatum succeeded to reduce the motor symptoms of HD [166]. The AAV mediated transfection of the RNA was extensively expressed throughout the striatum, demonstrating the efficiency of this approach [167]. shRNA methodology, however, poses the risk of overdose due to excessively strong promoters [168]. Intracellular overdose of shRNA can clog up micro-RNA the transcriptional cellular pathways, leading to potentially serious adverse effects. Therefore, the translation of shRNA methodology to the clinical settings is slowed down due to the necessity to improve the safety against shRNA-mediated toxicity [153]. The optimization of the potency of construct expression and promoter efficacy in the structures of interest is a translational challenge for shRNA [154]. The awarding of two Nobel Prizes for gene therapy-based discoveries for the last 14 years is an extremely strong indicator that this methodology is anticipated to be a game changer in the field of inherited disorders. The translation of RNA gene therapy requires further clinical trials to exclude: 1) cellular off-target effects, 2) intracellular overdose. The siRNA and shRNA techniques are likely to be translated in the next few years for the treatment of movement disorders but more tests are needed to establish the most optimal delivery and to ensure safety and tolerability of the RNAi-based gene therapy. Besides RNAi compounds there is another group of nucleotide-based molecules that induce RNA-dependent degradation to dispose of aberrant transcript, i.e. antisense oligonucleotides (ASO) (Fig. 3).

## 10 Therapeutic pole position for the antisense oligonucleotides

ASO are synthetic single-stranded nucleotides, with approximate length of 12–22 bases that complementary bind messenger RNA (mRNA) to supress mutant protein expression [169], [170]. One of the main advantages of ASO methodology compared to the other gene therapies is that single-stranded DNA diffuses well in the brain and is absorbed efficiently by the cells. The administration of ASOs into the cerebrospinal is widely distributed across the central nervous system. Intraventricular ASO injection was shown to promote the degradation of huntingtin mRNA and to repress the expression of mutant htt protein [171]. Such benefit allows the application of ASO gene therapy via the much less invasive lumbar injection, compared to the surgical intracranial administration of AAV [150]. The application of ASO was tested in preclinical trials for the treatment of inherited hypokinetic and hyperkinetic movement disorders.

The pathogenesis of Parkinson’s disease involves dominantly-inherited genetic causes. One of the most frequent loci of mutation is the leucine-rich repeat kinase 2 (LRRK2) and genetic errors or variations within the LRRK2 gene result in enhanced formation of abnormal protein, α-synuclein (aSyn). The neurotoxic aSyn accumulates intracellularly and leads to widespread neuronal degeneration. Recently-developed model for gene therapy of PD involves the administration of ASO to supress the post-transcriptional LRRK2 gene expression. Targeted delivery of ASO in mice brains inhibits local gene expression by directly binding to target mRNA and supressing the synthesis of aSyn [172]. Recent findings showed that ASO robustly lowered the LRRK2 levels, ameliorated disease-associated motor symptoms [173] and reversed disease phenotype in mouse models of PD [174]. The enormous potential of this methodology has led to clinical trials for the tolerability of LRRK2-induced ASO-based therapy in PD patients (ClinicalTrials.gov Identifier: NCT03976349). ASO is potential gene therapy for other genetic mutations involved in the pathogenesis of PD such as the SNCA gene. A recent study showed that ASO-based therapy is able to reduce production of aSyn in rodent models of PD [172]. The authors also addressed the translational potential of ASO through characterization of human SNCA targeting ASO. Their data revealed that ASO is able to suppress the human SNCA transcript in vivo and demonstrate the activity and distribution of SNCA-targeting ASO with corresponding decrease in aSyn cerebral spinal fluid (CSF) levels of non-human primates [172]. This funding suggests that ASO-targeting of the SNCA gene exhibits the potential to be a disease-modifying treatment for PD patients. A recent study also targeted SNCA with modified ASO with improved stability and cellular uptake in a mouse model of PD [174]. The authors confirmed that ASO efficiently downregulated SNCA at both the mRNA and protein level, and concurrently ameliorated neurological defects in the mice expressing human wild type SNCA [174]. Another neurological disorder is already undergoing clinical trials: ASO-based therapy is tested on patients with familial amyotrophic lateral sclerosis (ALS). Centrally delivered ASO therapeutics are in clinical phase 1 trials for familial ALS funded by Biogen and Ionis Pharmaceuticals. The clinical trials for ASO-based therapy targeting SOD1 (NCT02623699) and C9orf72 genes (NCT03626012) examine the safety, tolerability, pharmacokinetics and pharmacodynamics of ASO methodology in ALS patients [175], [176]. ASO was already successfully translated for the treatment of spinal muscular atrophy (SMA) with mutation of survival motor neuron 1 (SMN1) gene. Clinical trials demonstarted the safety, tolerability and efficacy of ASO-based gene therapy of SMA in humans [177], [178]. In 2016 the Food and Drug Administration and in 2017 and European Medicines Agency approved Nusinersen (trade name Spinraza; Biogen), a centrally delivered ASO drug, for SMA gene therapy [179]. Eteplirsen, another ASO-based medication, is approved for the treatment of different neuromuscular disorder: Duchenne muscular dystrophy (DMD) [180]. The robust translational development of ASO in the clinical settings for the treatment of neurodegenerative diseases and the ongoing clinical trials are reviewed by [181].

ASO has been proposed as a suitable treatment strategy for monogenic hyperkinetic movement disorders such as Huntington’s disease. Fundamental preclinical research showed that ASO robustly lowers the htt protein levels and ameliorates disease-associated symptoms and reverses disease phenotype in rodent models of HD [159], [171]. Current clinical 1b/2a trials funded by Wave Life Sciences (WVE-120101 and WVE-120102, NCT03225833) examined the safety and tolerability of ASO-based suppression of htt gene expression [182]. ASO-based methodology advanced one step further by supressing only the mutant form of htt protein. Seminal translational findings revealed the potential value of RNA-based technique: administration of ASO in the cerebrospinal fluid of HD patients reduced the levels of mutant htt [183], [184]. Application of ASO induced a dose-dependent decrease in the htt protein concentration four weeks after the start of the therapy of a clinical phase 1/2a trials funded by Funded by Ionis Pharmaceuticals and Hoffmann–La Roche (NCT02519036). In the first-in-human, double-blind clinical trial found significant, dose-dependent reductions of mutant htt protein measured in the cerebrospinal fluid [184]. The concentration of the mutant htt protein was lowered to 40–60% from the baseline levels [184]. The authors found no adverse effects and no patients were reported to be prematurely discontinued from treatment. Together, these findings reveal the proximity of ASO-based genetic technique to its clinical translation and the growing evidence for its imminent application in patients with HD. Another feature that makes ASO suitable for clinical application is ASO’s long half-lives in the nervous tissue [161]. In a mouse model of HD expressing the human htt gene, the htt mRNA is suppressed up to 12 weeks after discontinuation of the ASO-based therapy [171]. The lasting effect of ASO is confirmed in humans where ASO was detectable in the brain and spinal cord of ALS patients for 3 months after an administration of ASO in the cerebrospinal fluid [176]. ASO accumulate in the kidney where the metabolites are cleared in urine. This raised early concerns for ASO-based nephrotoxicity. A recent database, encompassing 32 clinical trials and 11 different ASO, showed no evidence of clinically significant renal dysfunction up to 52 weeks of randomized-controlled treatment [185]. Unlike other DNA and RNA techniques, ASO are not reported to trigger strong immunological response. The low cost of ASO delivery, effect longevity, negligible side effects, safety and tolerability altogether position currently ASO as the most feasible gene therapy for clinical translation [181]. The translation of ASO gene therapy requires: 1) evaluation of the degree of disease modifying effect, 2) evaluation of the long-term efficiency. These evaluations can be completed after longitudinal studies in patients undergoing gene therapy. ASO-based therapy is already approved for disorders, such as SMA and DMD. Based on the duration of ongoing clinical trials, the approval of ASO therapeutic agents for movement disorders, such as HD and PD, is expected to happen in the next few years.

## 11 Summary and outlook

Movement disorders encompass hypokinetic and hyperkinetic neurological conditions, requiring differential treatment approaches. In order to choose the most appropriate potential therapies we must consider their major methodological disadvantages (Table 1). A large portion of patients with PD and all patients with HD suffer from the products of aberrant genes that trigger the onset of the symptoms. A good candidate for gene therapy is movement disorders with dominantly-inherited genetic causes such as the htt gene for HD and LRRK2 or SNCA genes for PD. The tremendous advance of DNA-editing and RNA-based protein inactivation over the last few years is making the clinical implementation of gene therapy imminent. Among the genetic techniques ASO-based methodology is currently the most frequently approved and clinically tested gene therapy. However, shRNA and siRNA methodologies are progressing and successfully reducing the adverse effects, thus approaching clinical trials. CRISPR-Cas9 and zinc finger proteins are another likely candidate for clinical translation. Gene therapy will be expected to reduce or slow down the process of neurodegeneration in patients with familial movement disorders diagnosed in early stage. An early detection of movement symptoms, combined with genetic testing [186], [187] for the detection of an inherited aberrant gene would be needed for the initiation of gene therapy. Some of the movement disorders however are not inherited and others may result from genetic errors which are still not established. Part of these cases will rely on the developments of DBS stimulation particularly in the field of BCI and optogenetics. Electrical or optogenetic DBS will be also considered as a primary treatment choice for patients with movement disorders in advanced stage. While optogenetics is widespread in laboratory settings it needs further development for successful clinical translation. For patients with idiopathic movement disorders that are not suitable for DBS, the pharmacological therapy will be the predominant therapeutic approach, and these patients will be relying on the recent developments of immunotherapies and infusion therapy.

**Table 1:** Unresolved major methodological advantages (indicated with dark grey field) of the leading therapeutic candidates for movement disorders (references are in the main text).

## CRediT authorship contribution statement

**Marian Tsanov:** Conceptualization, Visualization, Writing - original draft, Writing - review & editing.

## Declaration of Competing Interest

The authors declare that they have no known competing financial interests or personal relationships that could have appeared to influence the work reported in this paper.
